# The evaluation and surgical management of post-intubation tracheal strictures at a thoracic surgery referral centre in South Africa

**DOI:** 10.7196/SARJ.2018.v24i2.193

**Published:** 2018-06-21

**Authors:** S Ramghulam, R Perumal, D Reddy

**Affiliations:** 1 Department of Cardiothoracic Surgery, School of Clinical Medicine, Nelson R Mandela School of Medicine, University of KwaZulu-Natal, Durban, South Africa; 2 Centre for the AIDS Programme of Research in South Africa, Nelson R Mandela School of Medicine, University of KwaZulu-Natal, Durban, South Africa; 3 Department of Pulmonology and Critical Care, University of Cape Town, South Africa

**Keywords:** trachea, post-intubation tracheal stenosis, tracheal reconstruction, tracheal dilatation

## Abstract

**Background:**

The surgical treatment of tracheal stenosis following endotracheal intubation or tracheostomy is well described in the developed world.

**Objectives:**

To describe our surgical experience with this pathology, and highlight the nuances of its diagnosis and management in South Africa.

**Methods:**

We reviewed the clinical records and archived imaging of all patients who underwent tracheal resection and reconstruction for post-intubation tracheal stenosis between 1 July 2003 and 31 July 2014 in the Department of Cardiothoracic Surgery at Inkosi Albert Luthuli Central Hospital, Durban, South Africa.

**Results:**

During the study period, 42 patients underwent tracheal resection. We evaluated the preoperative bronchoscopic characteristics of the tracheal stricture in all patients, and computed tomography (CT) was used as an adjunct in 28 (66%) patients. The stricture lengths determined by CT and intraoperative measurement were strongly correlated (*r* (27)=0.506, p=0.006), and the stricture lengths determined by bronchoscopy and intraoperative measurement were weakly correlated (*r* (41)=0.201, p=0.209). A total of 36 patients (85.7%) underwent surgery via a cervical approach and 6 (14.3%) via a right thoracotomy approach. There was no early mortality, and surgery was complicated by vocal cord palsy in 4 cases, restenosis in 2 cases, infection in 1 case and paraparesis in 1 case.

**Conclusion:**

Tracheal resection for the treatment of post-intubation tracheal stenosis can be undertaken safely with minimal complications in the developing world, with the vast majority of lesions approached via a cervical approach. A preoperative evaluation of the stricture using CT is an accurate technique for planning tracheal resection and reconstruction.

## Background

Despite progressive advances in critical care medicine with regard to invasive ventilation, tracheal strictures that follow endotracheal intubation or tracheostomy remain the most common non-malignant indication for tracheal resection and reconstruction worldwide.^[1,2]^

In 1996, Hermes Grillo stated that ‘any patient who has received ventilatory support in the recent past or even not-so-recent past, who develops signs and symptoms of upper airway obstruction, has an organic lesion until proved otherwise.’^[1]^ Despite the use of high-volume low-pressure endotracheal tube cuffs, the reported incidence of post-intubation tracheal strictures is estimated to be up to 11% in critically ill ventilated patients, and associated risk factors include systemic hypotension, long duration of ventilation and tracheostomy.^[2,3]^ It has also been suggested that tracheal stenosis following percutaneous tracheostomy occurs earlier than with surgical tracheostomy.^[4]^ The progression of symptoms of stridor in patients with an evolving tracheal stricture is variable, and often leads to misdiagnosis as asthma until the stridor is evidently not responsive to bronchodilator therapy, and the tracheal lumen significantly reduced to approximately 30% of the normal diameter.^[5]^ Spirometry generally illustrates the characteristic flow-volume loop appearance of fixed obstruction to maximal inspiration and expiration, whether intrathoracic or extrathoracic.^[6]^

Diagnostic investigations include plain chest radiography and computed tomography (CT) scanning of the tracheal air column, while bronchoscopy serves as the ideal evaluation of the trachea, as well as the glottis and vocal cords. Rigid bronchoscopy may also play a therapeutic role in dilating the area of stenosis, to temporarily relieve symptoms of stridor prior to definitive surgical management. When indicated, the surgical approach to a post-intubation tracheal stricture is based on the level and complexity of the lesion. The most common scenario encountered is an end-to-end resection with anastomosis, undertaken using a cervical approach, usually producing satisfactory results.

Despite the high incidence of post-intubation tracheal stenosis referred to tertiary thoracic surgery centres for tracheal resection, there have been no reports of the South African (SA) surgical experience with this pathology. All tracheal resections at our centre are undertaken by thoracic surgeons, as we are equally comfortable with both cervical and intrathoracic surgery, and tailor our approach to the patient’s pathology. This contribution highlights the nuances of diagnosing and treating this pathology in the context of a cardiothoracic surgical referral centre in SA.

## Methods

The Department of Cardiothoracic Surgery at Inkosi Albert Luthuli Central Hospital is the sole provider of cardiothoracic surgical care for the province of KwaZulu-Natal and the eastern seaboard of SA, encompassing a referral base of approximately 14 million patients. We undertook a retrospective cohort study of all patients with postintubation tracheal stenosis who underwent tracheal resection and reconstruction during the 11-year period from 1 July 2003 to 31 June 2014. The data were collected from electronic case records and perioperative imaging studies, including intraoperative photographic images. The study excluded patients with post-intubation tracheal stenosis treated by definitive endoscopic methods or surgical procedures other than tracheal resection and reconstruction.

All patients referred with suspected post-intubation tracheal stenosis underwent plain film chest radiography to exclude extrinsic airway compression and to evaluate the lung fields; rigid bronchoscopy was undertaken for diagnostic and therapeutic purposes. Computer tomography has been performed routinely in all patients since 2008, and prior to this, when clinically indicated. At bronchoscopy the vocal cords and the lesion were assessed, and the dimensions and nature of the tracheal stricture were documented to plan future surgical treatment. In patients with significant airflow obstruction, the tracheal stricture was progressively dilated using a series of paediatric and adult rigid bronchoscopes. This approach served to relieve symptoms and to allow time for further imaging modalities and the healing of associated injuries, and to assist with planning definitive surgical treatment on an elective basis. All patients with cicatricial concentric tracheal strictures were considered for definitive treatment by tracheal resection and end-to-end reconstruction. Associated comorbidities, including HIV infection, were managed by appropriate medical therapy. As a general principle, tracheal surgical intervention is viewed as the final planned operation in all patients, and this occasionally requires weeks to months of preparing patients with healing injuries such as burns or skin grafting, or patients requiring other surgical procedures in the interim, such as orthopaedic treatment of fractures.

**Fig. 1:**
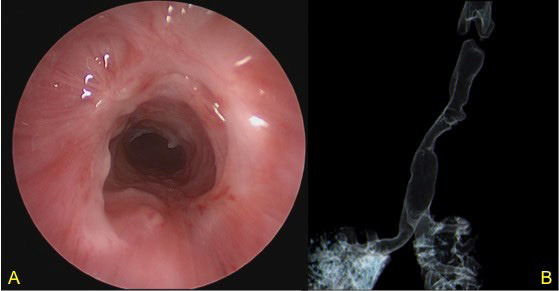
The two modalities used to evaluate post-intubation tracheal stenosis. A. The endoscopic appearance of a complex fibrous tracheal stricture that followed prolonged endotracheal intubation and tracheostomy. B. The reconstructed tracheal image from a CT scan, illustrating the length and location of the lesion in relation to the larynx and the carina. CT = computed tomography

The surgical approach to each patient was individualised based on the endoscopic data derived from rigid bronchoscopy, and CT was used to assist in surgical decision-making (Fig. 1).

**Fig. 2:**
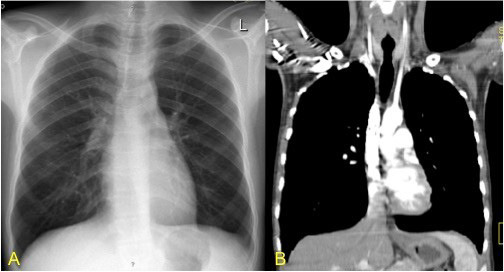
A. The postero-anterior plain chest radiograph and the coronal section of a CT scan. B. demonstrating a cervical post-intubation tracheal stricture distorting the tracheal air column. This lesion would be amenable to surgical treatment via the cervical approach. CT = computed tomography.

**Fig. 3:**
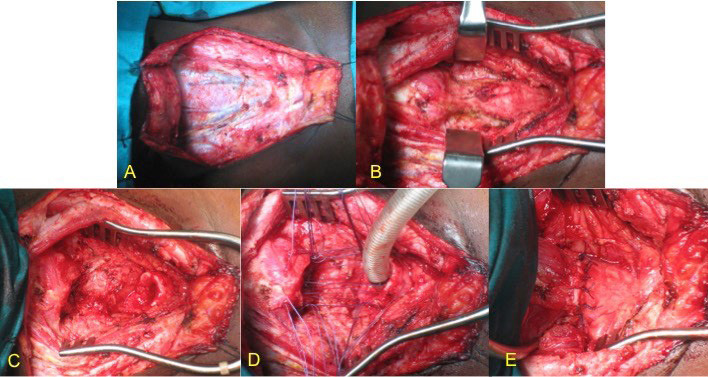
The intraoperative images for the patient depicted in Fig. 2. A. With the neck extended, a cervical collar incision is made and subplatysmal flaps are created. B. The 2 cm concentric fibrous stricture in the cervical trachea is exposed, with circumferential dissection restricted to the region of the stricture. C. The stricture is excised and the proximal and distal trachea are extensively mobilised, preserving the lateral vascular pedicles. D. Our preferred technique of cross-field ventilation while placing the interrupted, circumferential absorbable sutures prior to approximating the proximal and distal trachea. E. The reconstituted trachea with the neck in a flexed position to maintain a tension-free anastomosis.

**Fig. 4:**
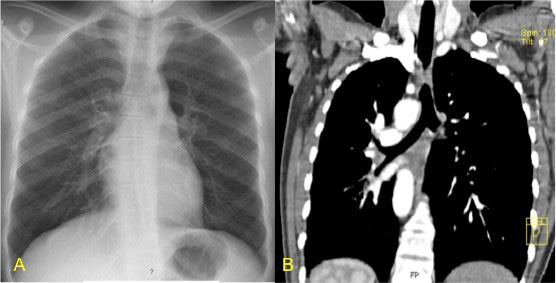
A. The postero-anterior plain chest radiograph, and B, the coronal section of a CT scan of a patient with an intrathoracic post-intubation tracheal stricture, which was resected via thoracotomy. CT = computed tomography

**Fig. 5:**
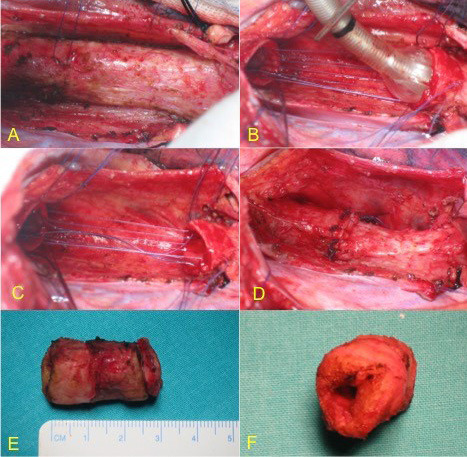
The intraoperative images of the patient depicted in Fig. 4. A. Via a right posterolateral thoracotomy, the azygous vein is divided and the mediastinal pleura opened, allowing access to the entire length of the intrathoracic trachea B. Following resection of the stenotic lesion, cross field ventilation of the left lung is achieved while interrupted absorbable sutures are placed circumferentially. C and D. The significant gap between the ends of the trachea is anastomosed in a tension-free fashion using hilar release techniques if required. E and F. The excised tracheal stricture with typical cicatricial features.

All patients underwent tracheal stricture resection and end-to-end reconstruction, either via a cervical approach (Figs 2 and 3) or via a thoracic approach (Figs 4 and 5), using techniques of tracheal reconstruction.

Median sternotomy and cardiopulmonary bypass were not employed in any patient, and the use of either a suprahyoid release procedure or a hilar release procedure was at the discretion of the surgeon, to ensure a tension-free anastomosis. All patients were maintained in neck flexion for at least 10 days after surgery, assisted by the use of a customised neck flexion harness and a chin-to-manubrium suture (guardian suture). Repeat bronchoscopy was not undertaken unless recurrence of symptoms necessitated intervention, and following hospital discharge, early outpatient follow-up was undertaken at 6 weeks and 6 months, and annually thereafter.

Data analysis was undertaken using SPSS software version 23.0 (IBM Corp., USA). For all statistical comparisons, a significance level of *p*<0.05 was used; correspondingly, 95% confidence intervals (CIs) were used to describe effect size. All data were assessed for normality, and nonparametric tests were used where necessary. Medians and interquartile ranges (IQRs) were used to describe data not amenable to parametric description. Pearson product-moment correlation coefficients were used to describe linear correlation between variables of interest.

The study was conducted under the oversight of the Biomedical Research Ethics Committee at the Nelson R Mandela School of Medicine (ref. no. BE468/14).

## Results

A total of 42 patients with tracheal stenosis were included in this study (Table 1), with a median follow-up of 114 (IQR 43 - 210) days. Twenty-nine patients were male (69%), and the mean (standard deviation; 95% CI) age was 22 (9.94) years. Significant preoperative laboratory parameters reflected a mean haemoglobin of 13.08 (1.41) g/L and a mean serum albumin of 42.6 (5.78) g/L.

**Table 1:** Preoperative patient characteristics

**Characteristic (N=42)**	
Age, mean (SD), years	22 (9.94)
Male, n (%)	29 (69)
Female, n (%)	13 (31)
Haemoglobin, mean (SD), g/L	13.08 (1.41)
Albumin, mean (SD), g/L	42.6 (5.78)
Reason for intubation: non-airway trauma, n (%)	42 (100)
Duration of ventilation, median (IQR), days	12 (8 - 21)
Duration to definitive surgery, median (IQR), days	49.5 (19.5 - 79.5)
Dilations before surgery, median (IQR), days	5.5 (2.75 - 9.00)

SD = standard deviation

IQR = interquartile range

The primary indication for intubation and ventilation in all 42 patients was trauma unrelated to the airway. The median duration of ventilation was 12 days (IQR 8 - 21), and the median time from presentation to definitive surgery was 49.5 days (IQR 19.5 - 79.5). Prior to tracheal resection, the median number of therapeutic tracheal dilations undertaken using rigid bronchoscopy was 5.5 dilations (IQR 2.75 - 9) per patient.

The tracheal stricture characteristics on bronchoscopy demonstrated a median (IQR) distance to the vocal cords of 40 (30 - 50) mm, median (IQR) stricture length of 20 (20 - 25) mm and median (IQR) distance to carina of 50 (40 - 60) mm (Table 2). The median (IQR) proportion of tracheal involvement was 18.2% (15.7 - 21.4). Computed tomography was performed in 28 cases, with the lesion characteristics determined by CT demonstrating a median (IQR) stricture length of 20 (14.25 - 25), and the median stricture length determined at surgery was 20 (20 - 25) mm. The stricture lengths determined by CT and intraoperative measurement were strongly correlated (r (27)=0.506; *p*=0.006). The stricture lengths determined by CT and bronchoscopy were moderately correlated (r (27)=0.472; *p*=0.01), and the stricture lengths determined by bronchoscopy and intraoperative measurement were weakly correlated (r (41)=0.201; *p*=0.209).

**Table 2:** Tracheal stenosis lesion characteristics

**Characteristic**	**Median (IQR), mm**
**Bronchoscopy (n=42)**	
Distance to vocal cords	40 (30 - 50)
Stricture length	20 (20 - 25)
Distance to carina	50 (40 - 60)
**Computed tomography (n=28)**	
Stricture length	20 (14 - 20)
**Intraoperative evaluation (n=42)**	
Stricture length	20 (20 - 25)

IQR = interquartile range

Upper tracheal lesions were resected via a cervical approach in 36 (85.7%) patients, with the remaining 6 (14.3%) with lower tracheal lesions undergoing right thoracotomy. In 2 cases a suprahyoid release was employed to ensure a tension-free anastomosis (Table 3). The median (IQR) period to discharge from definitive surgery was 10.5 (10 - 14) days. There was no perioperative mortality, and morbidity included vocal cord palsy in 4 patients, restenosis in 2 patients, infection in 1 patient and transient paraparesis in one patient.

**Table 3:** Operative technique and outcomes

**Characteristic (N=42)**	***n *(%)**
**Surgical approach**	
Cervical	36 (85.7)
Thoracic	6 (14.3)
**Suprahyoid release**	2 (4.8)
**Guardian stitch, n**	42 (100)
**Duration to discharge, median (IQR), days**	10.5 (10 - 14)
**Complications**	
Vocal cord paralysis	4 (9.5)
Restenosis	2 (4.8)
Infection	1 (2.4)
Paraparesis	1 (2.4)

IQR = interquartile range.

## Discussion

We have described a cohort of patients with post-intubation tracheal stenosis treated by tracheal resection, the majority of whom were male (69%) and young (mean age 22 years), where the primary indication for intubation was predominantly trauma unrelated to the airway. The median duration of ventilation of 12 days is consistent with the need for adjuvant surgical procedures, including relook laparotomies in patients with an open abdomen, and recovery following head injuries.

Plain chest radiography remains the primary imaging modality for patients with suspected post-intubation tracheal strictures, often to examine the lung fields, as impaired clearing of secretions may result in bronchopneumonia. Obscuration of the tracheal air column may be noted, but the absence of this feature is not helpful. Following plain radiographic evaluation, rigid bronchoscopy is a key component in the primary diagnostic and therapeutic management of patients with post-intubation tracheal strictures. Although bronchoscopy remains the gold standard for the identification and characterisation of airway lesions, it fails to provide adequate visualisation of the post-stenotic trachea in cases of severe luminal narrowing. In addition, bronchoscopy is an invasive procedure that may cause patient discomfort, requires sedation and is associated with a 0.8% morbidity.^[8]^ Of critical value is the therapeutic role of rigid bronchoscopy in dilating severely stenotic lesions, as a resuscitative manoeuvre prior to intubation, or as a temporising measure while surgery is being planned. In rare instances, tracheal dilatation of an early stricture may suffice as definitive therapy, without the need for late intervention. However, limited direct control of bleeding distal to the lesion is possible, following diagnostic or interventional bronchoscopy. In this study, patients required a median of 5.5 serial tracheal dilatations prior to definitive surgical therapy, with surgery only undertaken once all associated injuries and open wounds were healed. In addition to assessing vocal cord function and obtaining bronchoalveolar lavage specimens, detailed evaluation of the tracheal stricture is made, including the nature (fibrous v. granulation tissue), the configuration (concentric v. eccentric) and the diameter, length and location of the stricture/s. Despite the widespread use of bronchoscopy as a tool to determine stricture length, we found that the overall correlation with findings at surgery were poor. This may be related to operator/observer variability, or the fact that strictures may evolve with repeated dilatation, resulting in the external scarring noted at surgery being inconsistent with the length of the intraluminal lesion measured at bronchoscopy.^[8]^

CT was performed to improve the accuracy of our evaluation of tracheal stricture length and location in patients with tracheal strictures necessitating surgical intervention, with a specific interest in evaluating the airway using virtual bronchoscopy airway reconstruction techniques.^[9]^ CT is a non-invasive procedure and well tolerated by all patients. Taha *et al*.^[10]^ reported that the detection rate of post-intubation tracheal stenosis was 94% by CT, and 88% by rigid bronchoscopy, and CT provided additional information on the morphology of the tracheal wall. Tracheostomy tubes were removed prior to chest CT, thereby avoiding radiographic artifacts, and high-resolution imaging of the lung fields allowed exclusion of any chronic pulmonary sequelae in patients with a prolonged ventilatory course. The non-invasive nature of this imaging modality made it complementary to rigid bronchoscopy, and a strong correlation between the evaluation of tracheal stricture length on CT and findings at surgery was found.^[11]^ The combined use of CT and endoscopic evaluation has allowed for extremely accurate preoperative planning, including the surgical approach, the need for adjunctive release manoeuvres and the prediction of postoperative complications.

The definitive surgical treatment of short segment tracheal strictures is resection with end-to-end anastomosis.^[12]^ While an approach involving primary emergency tracheal resection upon diagnosis may be favoured by some centres,^[13]^ it is widely held that tracheal reconstruction should be the final act in foreseeable tracheal instrumentation. The median duration from presentation to definitive surgery of approximately 7 weeks in this study generally reflected the need for optimisation of patients prior to definitive surgery. For example, one patient with extensive burns to the chest and face underwent multiple reconstructive surgeries during his 20-week admission prior to tracheal resection, during which time his stridor was palliated by weekly dilatation of the tracheal stricture with rigid bronchoscopy. Similarly, patients with open wounds underwent skin grafting prior to definitive tracheal surgery.

The core principles of tracheal resection and reconstruction are well established, and the surgical technique was consistent across all surgeons in this study.^[14,15]^ The vast majority of patients presented with tracheal strictures in the upper trachea, as seen at bronchoscopy and confirmed on CT scan, and therefore underwent tracheal resection and end-to-end reconstruction via a cervical collar incision. In six patients the stricture was situated in the lower trachea, necessitating an approach via right posterolateral thoracotomy. Following tracheal dilatation via rigid bronchoscopy, oral intubation is performed with a single lumen reinforced endotracheal tube, with cross-field ventilation maintained by the surgeon after division of the trachea. The tracheal anastomosis is undertaken with interrupted 3-0 Vicryl sutures (Ethicon Inc., USA). A tension-free anastomosis is ensured by maintaining the patient’s neck in a flexed position, aided by a suture from chin to manubrium (guardian stitch). In a minority of patients, additional release manoeuvres are necessary, the most common being a suprahyoid release, as described by Montgomery.^[16]^ All patients are extubated after the procedure and remain in a neck flexion position for 10 days, and are discharged after release of the guardian suture. The complications encountered after tracheal resection are well described, with four patients in this study having experienced varying degrees of laryngeal dysfunction, with unilateral vocal cord palsy. Both the patients who underwent suprahyoid release complicated with vocal cord palsy, which is known to be a risk of this technique.^[16]^ Two patients presented with restenosis after tracheal resection. In one patient, the cause was granulation tissue at the anastomosis, and this was dealt with by rigid bronchoscopy, with no subsequent recurrence. The second patient had undergone long-segment tracheal resection following tracheostomy-site stenosis, and after a second attempt at tracheal resection, she was ultimately managed with Montgomery T-tube insertion. Previous tracheostomy, long-segment tracheal resection and prior tracheal resection are documented risk factors for surgical failure and postoperative complications.^[17–19]^ Montgomery T-tube placement affords normal airway humidification and speech, and less damage to the stomal area than with tracheostomy.^[20]^ An alternative is the use of a fenestrated tracheostomy tube, which has similar benefits.^[21]^ In exceptional circumstances, tracheal stents may be considered to manage complex lesions, but the associated complications of stent migration and mucosal overgranulation limit their effectiveness.^[22,23]^ Other complications encountered included superficial cervical wound infection, and transient paraplegia probably related to hyperflexion of the neck in the postoperative period.^[24,25]^

The outcomes of tracheal resection for post-intubation tracheal stenosis in this study are consistent with those of other published series.^[26,27]^ Ventilation for trauma in young patients remains a significant cause of post-intubation tracheal stenosis, and such patients often require adjuvant surgical procedures prior to definitive airway surgery. This study suggests that CT scan is an accurate preoperative investigation method to estimate the length of the segment to be excised, although rigid bronchoscopy remains a cornerstone of early management. A combination of endoscopic and CT imaging allows the surgical approach to be planned, but ultimately the intraoperative findings upon transection of the trachea dictate the technique of reconstruction and the potential need for adjunctive release procedures. Utilising standard principles of tracheal resection and end-to-end reconstruction as described by others, successful outcomes were achieved in 95.2% of patients undergoing surgery.
